# Temporal Pattern of Neuroinflammation Associated with a Low Glycemic Index Diet in the 5xFAD Mouse Model of Alzheimer’s Disease

**DOI:** 10.1007/s12035-022-03047-3

**Published:** 2022-09-29

**Authors:** Ioannis Dafnis, Christina Mountaki, Eleni Fanarioti, Dimitrios C. Mastellos, Michalis Karvelas, Vaios T. Karathanos, Athina Tzinia, Catherine R. Dermon, Angeliki Chroni

**Affiliations:** 1 Institute of Biosciences and Applications, National Center for Scientific Research “Demokritos”, Agia Paraskevi, Athens Greece; 2 Department of Biology, Human and Animal Physiology Lab, University of Patras, Patras, Greece; 3 Institute of Nuclear & Radiological Sciences and Technology, Energy & Safety, National Center for Scientific Research “Demokritos”, Agia Paraskevi, Athens Greece; 4 Research and Development Department, Agricultural Cooperatives’ Union of Aeghion, Aeghion, Greece; 5 Laboratory of Chemistry-Biochemistry-Physical Chemistry of Foods, Department of Dietetics and Nutrition, Harokopio University, Kallithea, Greece

**Keywords:** Alzheimer’s disease, Low glycemic index diet, Microglia, Neuroinflammation, TNFα

## Abstract

Alzheimer’s disease (AD) is associated with brain amyloid‐β (Aβ) peptide accumulation and neuroinflammation. Currants, a low glycemic index dried fruit, and their components display pleiotropic neuroprotective effects in AD. We examined how diet containing 5% Corinthian currant paste (CurD) administered in 1-month-old 5xFAD mice for 1, 3, and 6 months affects Aβ levels and neuroinflammation in comparison to control diet (ConD) or sugar-matched diet containing 3.5% glucose/fructose (GFD). No change in serum glucose or insulin levels was observed among the three groups. CurD administered for 3 months reduced brain Aβ42 levels in male mice as compared to ConD and GFD, but after 6 months, Aβ42 levels were increased in mice both on CurD and GFD compared to ConD. CurD for 3 months also reduced TNFα and IL-1β levels in male and female mouse cortex homogenates compared to ConD and GFD. However, after 6 months, TNFα levels were increased in cortex homogenates of mice both on CurD and GFD as compared to ConD. A similar pattern was observed for TNFα-expressing cells, mostly co-expressing the microglial marker CD11b, in mouse hippocampus. IL-1β levels were similarly increased in the brain of all groups after 6 months. Furthermore, a time dependent decrease of secreted TNFα levels was found in BV2 microglial cells treated with currant phenolic extract as compared to glucose/fructose solution. Overall, our findings suggest that a short-term currant consumption reduces neuroinflammation in 5xFAD mice as compared to sugar-matched or control diet, but longer-term intake of currant or sugar-matched diet enhances neuroinflammation.

**Supplementary Information:**

The online version contains supplementary material available at 10.1007/s12035-022-03047-3.

## Introduction

Late-onset Alzheimer’s disease (AD), the most common neurodegenerative disease, affects millions of people worldwide. AD is characterized neuropathologically by the progressive appearance of extracellular plaques composed predominately of amyloid‐β (Aβ) peptide and of intraneuronal tangles of hyperphosphorylated tau [1, 2]. In addition, brain inflammation, that can be exacerbated by aggregated proteins, plays a significant role in neurodegeneration and progression of AD [3, 4]. Chronic activation of inflammatory pathways has been proposed to be either the consequence of or the driving force for AD pathogenesis [3–5]. Several inflammatory mediators, including cytokines, complement proteins, chemokines, cyclooxygenase and acute phase proteins, are produced by microglia, astrocytes, and neurons and contribute to neuroinflammatory responses in AD [3–5]. Immune and inflammatory mediators, such as tumor necrosis factor α (TNFα), interleukin-1β (IL-1β), IL-6, IL-12, IL-23, interferon-γ (IFN-γ), complement protein C3, granulocyte–macrophage colony-stimulating factor, cyclooxygenase-2, and NF-κB1 have been found upregulated in animal models of AD or in brain or cerebrospinal fluid (CSF) from humans with AD [3, 4, 6]. Furthermore, meta-analyses of observational and epidemiological studies suggested that AD is accompanied by dysregulation of inflammatory markers, including blood and CSF cytokines, and this dysregulation is associated with an increased risk of all-cause dementia [7]. Moreover, a chronic low-grade inflammation has been associated with earlier disease onset in carriers of the apolipoprotein E (apoE) ε4 allele, which is the major genetic risk factor for late-onset AD [8]. The role though of chronic inflammatory events in worsening or exposing AD-specific degenerative processes remains relatively under-studied, and little is known about how the period of exposure affects AD-related pathogenetic events.

Studies of cognitive function and analyses of changes in neuroimaging and CSF biomarkers have suggested that a preclinical phase of AD precedes the onset of clinical symptoms by at least 10–20 years [9]. This phase is characterized by early deposition of Aβ in cortical regions along with early neuroinflammatory changes (such as microglial activation), followed by accumulation of tau pathology and cortical and hippocampal volume loss that lead to neurodegeneration and onset of symptomatic cognitive impairment [9]. Despite great efforts for the development of AD-targeted therapeutics, currently, there is no cure or effective treatment for slowing down the disease progression. A great amount of research has been also focused on finding potentially modifiable risk factors for AD, such as specific types of diet that could affect the disease progression [10, 11] and it has been suggested that initiation of appropriate dietary intervention early in disease progress could be most effective [11]. Over the last years, several reports have highlighted the protective effects of grapes and raisins, that are dried grapes, against AD in in vivo and cell-based studies. Specifically, consumption of a grape formulation (freeze-dried grape powder) twice a day for 6 months was found to be protective against significant metabolic decline in regions of the brain known to be affected in the early stages of AD in a study of people with mild cognitive decline [12]. Grape powder consumption also produced beneficial effects in rats fed a high-fructose–high-fat diet, exhibiting the potential to ameliorate changes in neurodegeneration-related proteins in the brain [13]. Furthermore, consumption of currants (black-colored raisins) for 60 days was shown to protect against spatial memory impairment and oxidative stress in a rat model of AD that was induced by intraperitoneal injection of aluminum chloride [14]. A recent study from our lab showed that the polar phenolic extract from Corinthian currants has a beneficial effect against oxidative stress induced by the cellular uptake of Aβ42 in the presence of apoE4 pathogenic forms in human neuroblastoma SK-N-SH cells [15]. The prevention of cellular redox status changes in the presence of the polar phenolic currants extract could be attributed to antioxidant free radical-scavenging properties [15].

Raisins are consumed worldwide and contain beneficial components for human health, such as phenolic compounds [16]. Raisins varieties include dark raisins, golden raisins, sultanas, and Corinthian currants [16]. Raisins have been shown to possess a low-to-moderate glycemic index and this makes them a healthy snack [16]. Raisin intake has been shown to lower inflammatory cytokines in human plasma [17], while phenolic extracts of raisins prevented inflammatory responses in human colon cancer cells [18] and gastric adenocarcinoma epithelial cells [19]. Raisins produced from a special type of black grape, i.e., *Vitis Vinifera L.*, var. Apyrena, are designated as Corinthian currants and are produced almost exclusively in Southern Greece, while those designated as Vostizza currants correspond to a high quality category [20]. Corinthian currants are rich in polyphenols and anthocyanins [15, 20, 21], compounds known to prevent oxidative stress which is associated with inflammation and also to affect cell signaling, transcription, and translation related to inflammation [22]. Additionally, several polyphenolic compounds of Corinthian currants (quercetin, resveratrol, kaempferol, and epigallocatechin gallate) have been shown to display anti-amylogenic and neuroprotective effects, including inhibition of Aβ oligomer formation, reduction of Aβ-stimulated apoptosis in neuronal cultures, and inhibition of Aβ-induced NF-κβ activation [23–26]. The neuroprotective effect of polyphenols has been found to be exerted by modulation of signaling pathways, such as the PI3K/Akt, ERK/CREB/BDNF, and GSK-3 cascades [25–27].

In the current study, we examined the effect of dietary supplementation with Corinthian currants on Aβ42 levels and inflammatory responses using the 5xFAD transgenic mouse model of AD. This AD mouse model exhibits intraneuronal Aβ42 accumulation at 1.5 months, amyloid deposition in the cortex and subiculum at 2 months of age, as well as neuroinflammation, demonstrated by astrogliosis and microgliosis, that begins at approximately the time when amyloid deposition initially appears [28]. We initiated the diet intervention at an early stage of the disease (1-month-old mice) and examined the effect of currant-supplemented diet, administered for 1, 3, and 6 months, in comparison to normal diet or sugar-matched diet on Aβ42, cytokine, and other AD-related protein levels. Our analysis suggests that a short-term currant consumption reduces Aβ42 levels and inhibits neuroinflammation in 5xFAD mice, but longer-term intake diminishes the protective effect.

## Materials and Methods

### Experimental Animals

The 5xFAD mice on a C57BL/6 background co-express and co-inherit familial AD mutant forms of human amyloid precursor protein (APP) [Swedish (K670N, M671L), Florida (I716V) and London (V717I) mutation] and presenilin-1 (M146L and L286V) transgenes under the transcriptional control of the neuron-specific mouse Thy-1 promoter [28, 29]. 5xFAD mice were kept in a heterozygote state and genotyping was performed by PCR analysis of tail DNA using primers specific for human APP gene (forward 5′-GAATTCCGACATGACTCA-3′, reverse: 5′-GTTCTGCTGCATCTTGGACA-3′) [30]. PCR reactions were performed for 30 cycles at 95 °C for 1 min, 60 °C for 1 min, and 72 °C for 1 min. Animals were kept under standard conditions (24 °C, 12-h light/dark cycle, lights on at 8:00 AM) and received food and water ad libitum. All animal procedures were performed in compliance with European legislation and were approved by the Animal Care and Use Committee of the NCSR “Demokritos” and by the Directorate of Agricultural and Veterinary Policy of the Region of Attica (Animal welfare assurance number: EL25 BIO 039, 6806/21–12-2018).

### Currants Paste and Extract

Currants paste was prepared, as described [31], using Corinthian currants produced from a special type of black grape, i.e., *Vitis Vinifera L.*, var. Apyrena, from the sub-area of Aeghion (Vostizza), Peloponnese, Southern Greece, and was provided by the Agricultural Cooperatives’ Union of Aeghion, Greece. Briefly, currants were loaded into a cutter machine (Recoscreen DC.200, CFS) that permits the mechanical separation of undesirable seeds from the final product. The operating principle is based on the transfer of currant paste by means of a vane pump (feed screw speed 16 rpm), through an extruder type device, which forces the product to pass through a system of sieves (filter screw speed 93 rpm). Currant paste was stored at 25 °C, packaged in hermetic polyethylene containers until further analysis.

To obtain the polar phenolic extract from currants, freeze dried currants were treated with methanol, as described [15, 32]. Aliquots of methanolic extract were kept under nitrogen at − 20 °C. Total polar phenolic content of the extract was assessed by the Folin Ciocalteu assay [33], using gallic acid as the reference standard. The milligrams of gallic acid equivalents (GAE) per milliliter of methanolic extract are used for the expression of currant extract concentration. The predominant sugars in Corinthian currants are fructose and glucose, at almost equal amounts, and account for approximately 65–70% of their weight [34]. Glucose concentration in currant extract was measured by the glucose oxidase–peroxidase method using the commercially available reagent Glucose LS (LABKIT Chemelex SA).

### Experimental Design

Male and female transgenic animals aged 4 weeks were randomly divided into nine groups that received three types of diet for 1, 3, and 6 months (in total: 99 mice with 6 male and 5 female per diet and time point). Specifically, mice were fed (a) standard chow diet, (b) chow diet containing 5% (w/w) currants paste, and (c) chow diet containing 1.75% (w/w) glucose/1.75% (w/w) fructose that is sugar-matched for the sugar content in currants [34]. For the incorporation of currants paste and glucose/fructose into mouse chow, the pellets were grinded into powder in a blender and then mixed with appropriate amount of currants paste or glucose/fructose and 0.65% (w/w) water. Standard diet was also grinded into powder and mixed with water. New pellets were shaped by hand and allowed to dry for 6 h in room temperature before provided to mice. Fresh diet was prepared every 2–3 days.

At baseline, mice were weighted and blood was drawn from the tail vain. At the end of each feeding period, mice were also weighted and then sacrificed by decapitation. Approximately 1 mL of blood was collected per animal by cardiac puncture using 26G 3/8 needles (BD Biosciences) into regular 1.5-ml Eppendorf tubes. Blood was kept at room temperature for 1 h to allow blot clotting and then the serum was collected at the supernatant following centrifugation of samples for 15 min at 1500 × *g* at 4 °C and stored in aliquots at − 80 °C. Furthermore, after mouse decapitation, each brain was removed, washed with ice cold phosphate-buffered saline (PBS), and hemisected. The cortical region was dissected out from one hemisphere and along with the other hemisphere were flash frozen in liquid nitrogen. Cortex was stored for biochemical analyses and hemisphere for immunohistochemical analyses at − 80 °C.

### Serum Analyses

Serum glucose and cholesterol levels were determined using the commercially available reagents Glucose LS and Cholesterol LS (LABKIT Chemelex SA), respectively. Serum insulin concentration was measured using the mouse insulin ELISA kit (EMD Millipore Corporation).

### Cortex Processing

Each cortex was homogenized with fifteen strokes using a glass-Teflon homogenizer in 500 μl Buffer A (50 mM Tris, 150 mM NaCl, containing 20 mM NaF, 1 mM Na_3_VO_4_, 1 mM 4-(2-aminoethyl)benzenesulfonyl fluoride hydrochloride, complete mini protease inhibitor cocktail (Roche), and phosphatase inhibitors PhosSTOP (Roche), pH 7.5). A total of 50 μl of homogenized cortex samples were diluted with 150 μl of buffer A and were further homogenized by sonication (Ultrasonic Processor UP200S, Heilscher Ultrasonics, Teltow) with a 3-mm diameter probe at 50% per second pulse mode and 30% sonic power, for 20 s × 3 with 40-s intervals between sonication periods. Then, the samples were stored as homogenate fractions at − 80 °C. The remaining 450 μl of each homogenized cortex sample, following glass-Teflon homogenization, were centrifuged at 50,000 × *g* for 25 min at 4 °C and each supernatant was stored as soluble fraction at − 80 °C until use. Pellet was resuspended via sonication in 500 μl Buffer B (50 mM Tris, 150 mM NaCl containing 20 mM NaF, 1 mM Na_3_VO_4_, 1 mM 4-(2-aminoethyl)benzenesulfonyl fluoride hydrochloride, Complete Mini Protease Inhibitor Cocktail, phosphatase inhibitors PhosSTOP, 0.5% (w/v) sodium deoxycholate, 1% (v/v) Triton × 100, 1% (w/v) SDS, pH 8.0). After centrifugation at 50,000 × *g* for 25 min at 4 °C, supernatant was saved as detergent-soluble fraction at − 80 °C until use. The protein levels of the homogenate, soluble, and detergent-soluble fractions were quantified using a BCA protein assay kit (Pierce).

### Cell Cultures

BV2, murine microglial cells, were maintained at 37 °C and 5% CO2 in a humidified incubator. Cells were cultured in RPMI 1640 Media (Biowest) supplemented with 10% fetal bovine serum (Biosera) and 100 U/ml penicillin G and 100 mg/ml streptomycin sulfate. BV2 cells were plated on 24-well plates at a density of 3 × 10^4^ cells per well in RPMI complete medium. The next day, the cells were incubated in the presence or absence of Corinthian currant extract or a glucose/fructose mixture in serum-free medium for 24 and 48 h at 37 °C. The currant polar phenolic extract was used at concentrations 1 and 5 μg GAE/mL and the glucose/fructose mixture at concentrations 2.6 and 12.6 mM that match the glucose/fructose concentration of currant extract. The final methanol concentration in each well was 0.68% (v/v). The cell medium was collected after indicated treatments and supplemented with Complete Mini Protease Inhibitor Cocktail and 1 mM serine protease inhibitor 4-(2-aminoethyl)-benzenesulfonyl fluoride. The cells were washed with ice-cold PBS and lysed at 4 °C in lysis buffer (50 mM Tris–HCl (pH 7.5), 150 mM NaCl, 1% (v/v) Nonidet P-40, 0.25% (w/v) deoxycholate, and Complete Mini Protease Inhibitor Cocktail). The protein concentration in cell lysates was determined using the Lowry assay (DC Protein Assay Kit, Bio-Rad).

### ELISA

Aβ42 levels were measured in detergent-soluble fractions (15 μg of total protein) using the Aβ42 Quantikine ELISA Kit (R&D Systems) according to the manufacturer’s guidelines. TNFα and IL-1β levels were measured in homogenate fractions (150 μg of total protein) using the mouse TNF-alpha DuoSet ELISA kit (R&D Systems) and mouse IL-1 beta ELISA kit (R&D Systems), respectively, according to manufacturers’ instructions. TNFα levels in BV2 cell medium after incubation of cells with currant extract or glucose/fructose mixture were also measured by the mouse TNF-alpha DuoSet ELISA kit (R&D Systems).

### Western Blot Analysis

For measurement of phosphorylated Akt, STAT3, and GSK3β, 75 μg of total protein in detergent-soluble fractions were loaded per lane on 9% Tris–glycine gels. For measurement of levels of total apoE and apoE fragments, 100 μg of total protein in detergent-soluble fractions were loaded per lane on 15% Tris–glycine gels. For measurement of levels of sAPPα and BDNF, 75 μg of total protein in soluble fractions were loaded per lane on 13% Tris–Glycine gels. After SDS-PAGE, proteins were transferred onto nitrocellulose membranes for immunoblotting using the following primary antibodies according to manufacturers’ instructions: rabbit anti-phospho-Akt (Ser473) monoclonal antibody D9E (1:2000, Cell Signaling Technology); rabbit anti-phospho-Stat3 (Tyr705) monoclonal antibody D3A7 (1:2000, Cell Signaling Technology); rabbit anti-phospho-GSK-3-beta (Ser9) monoclonal antibody 5B3 (1:1000, Cell Signaling Technology); goat anti-mouse apoE polyclonal antibody M-20 (1:500, Santa Cruz Biotechnology); mouse anti-β-amyloid monoclonal antibody 6E10 (1:600, BioLegend); rabbit anti-BDNF polyclonal antibody N-20 (1:500, Santa Cruz Biotechnology); rabbit anti-β-actin monoclonal antibody 13E5 (1:1000, Cell Signaling Technology). For analysis of phosphorylation of Akt, STAT3 and GSK3β, the membranes were stripped using stripping buffer (Thermo Fisher Scientific) and subsequently re-probed for the detection of total Akt using the rabbit anti-Akt (pan) monoclonal antibody C67E7 (1:1000, Cell Signaling Technology), total STAT3 using the rabbit anti-STAT3 monoclonal antibody 79D7 (1:2000, Cell Signaling Technology) and total GSK3β using the rabbit anti-GSK3β monoclonal antibody 27C10 (1:1000, Cell Signaling Technology). Following incubation of membranes with the appropriate secondary antibodies [goat anti-rabbit IgG coupled with horseradish peroxidase (1:2000, Merck Millipore), goat anti-mouse IgG coupled to horseradish peroxidase (1:2000, Merck Millipore) or rabbit anti-goat IgG coupled with horseradish peroxidase (1:3000, Dako)], blots were visualized with an enhanced chemiluminescent horseradish peroxidase substrate (Thermo Fisher Scientific). Quantification of protein levels was performed by densitometry of the immunoreactive bands by ImageJ software. pAkt, pSTAT3, and pGSK3β levels were normalized to their respective total Akt, total STAT3 and total GSK3β levels. ApoE, sAPPα, and BDNF levels were normalized to β-actin levels.

### Immunohistochemistry

Coronal sections of frozen hemispheres from male mice, 20 μm thick, were cut in a cryostat Leica (CM1500), thaw-mounted on gelatin-chromalum-coated glass slides and stored at − 75 °C, until experiments were performed. At the first experimental day, sections were post-fixed for 15 min with freshly prepared ice-cold 4% (v/v) paraformaldehyde in 100 mM PBS, pH 7.4, rinsed with PBS, treated with 0.3% (v/v) H_2_O_2_ to block endogenous peroxidase and blocked with 5% (w/v) BSA and 1% (v/v) normal horse serum (NHS) in PBS containing 0.05% (v/v) Tween-20 (PBS-T). Subsequently, sections were incubated overnight at 4 °C with rabbit polyclonal anti-TNFα (1:200, NBP1-19,532, Novus Biologicals) diluted in PBS-T with 1% (w/v) BSA and 0.15% (v/v) NHS. After washing in PBS, the sections were incubated with a biotinylated horse anti-rabbit IgG secondary antibody (R.T.U., BP-1100–50, Vector Laboratories) for 50 min at room temperature. The sections were then incubated with the reagents of Vectastain ABC-HRP kit (Vector Laboratories) for 1 h, washed, treated with reagents of diaminobenzidine (DAB) Substrate kit (Pierce) for approximately 10 min, rinsed with PBS, dehydrated, and coverslipped with Entellan rapid mounting medium (Merck).

### Microscopic Analysis and Quantification

For the determination of TNFα immunoreactive cells, 10 sections × 3 graduated frames of 100 μm^2^ per section of hippocampus were quantified manually at × 400 magnification with the aid of camera lucida attached to a light microscope (Optiphot-2μ Nikon). The investigator performing the quantification was blinded to the treatment groups. The TNFα-expressing cells were determined for CA1, CA2, CA3, and DG hippocampal layers according to the Paxinos and Franklin mouse brain atlas [35]. Images were acquired using Nikon Eclipse E800 microscope (× 400) connected to a PC and visualized by the Image J image analysis software (NIH, Bethesda, MD, USA).

### Double Immunofluorescence Labelling

Double-labeling experiments were performed to determine the co-localization of TNFα with CD11b integrin (clone OX-42) that in brain its expression is restricted to microglia. For this, mouse hippocampal sections were post-fixed (as described in the immunohistochemistry section) and following antigen retrieval, sections were blocked with 5% (w/v) BSA and 1% (v/v) NHS in PBS-T and incubated for 18 h at 4 °C with a cocktail of a combination of primary antibodies for TNFα (rabbit polyclonal, 1:200, NBP1-19,532, Novus Biologicals) and CD11b (mouse monoclonal OX-42; 1:200; MA1-81,606, Invitrogen) in PBS-T buffer with 1% (w/v) BSA and 0.15% (v/v) NHS. Secondary antibodies, donkey anti-rabbit IgG Alexa Fluor 488 (1:400, Invitrogen) and donkey anti-mouse IgG Alexa Fluor 555 (1:400, Invitrogen) diluted in PBS-T with 1% (w/v) BSA and 0.15% (v/v) NHS, were used simultaneously for 2 h and 30 min at room temperature in the dark. The sections were rinsed extensively with PBS and distilled water and coverslipped with the Vectashield hardset antifade mounting medium (Vector Laboratories). No labeling was observed in the absence of secondary antibodies, in a series of experiments performed in adjacent sections.

### Confocal Microscopy

Double immunofluorescent staining observation was performed with a Leica TCS SP8 (Leica Microsystems CMS GmbH) confocal microscope, using the × 63 objective. Images were acquired, maintaining fixed laser intensities and camera settings, with LAS X software (Leica Microsystems CMS GmbH). Density measurements of the double-labeled cells were quantified manually, blindly to the treatment group, and determined the population of TNFα + cells that co-expressed the microglial CD11b, clone OX-42, per hippocampal sub-region (as determined by the LasX software).

### Statistical Analysis

Quantitative data are presented as mean ± SD. Statistical analyses were conducted using the GraphPad Prism software and IBM SPSS Statistics software according to the type of data. Statistical comparisons between two groups were analyzed for significance by unpaired two-tailed Student’s *t*-test. For data obtained following immunohistochemistry the statistical comparisons were performed using a two-way analysis of variance (ANOVA) followed by post-hoc Tukey comparisons or independent t-test as required, with independent variables the “time of treatment” and “type of diet.” Differences were considered significant at *p* < 0.05. *p* values for statistically significant differences are indicated in each figure legend.

## Results

### Currant Diet Has no Effect on Body Weight and Serum Cholesterol, Glucose, and Insulin Levels of 5xFAD Mice

**Fig. 1:**
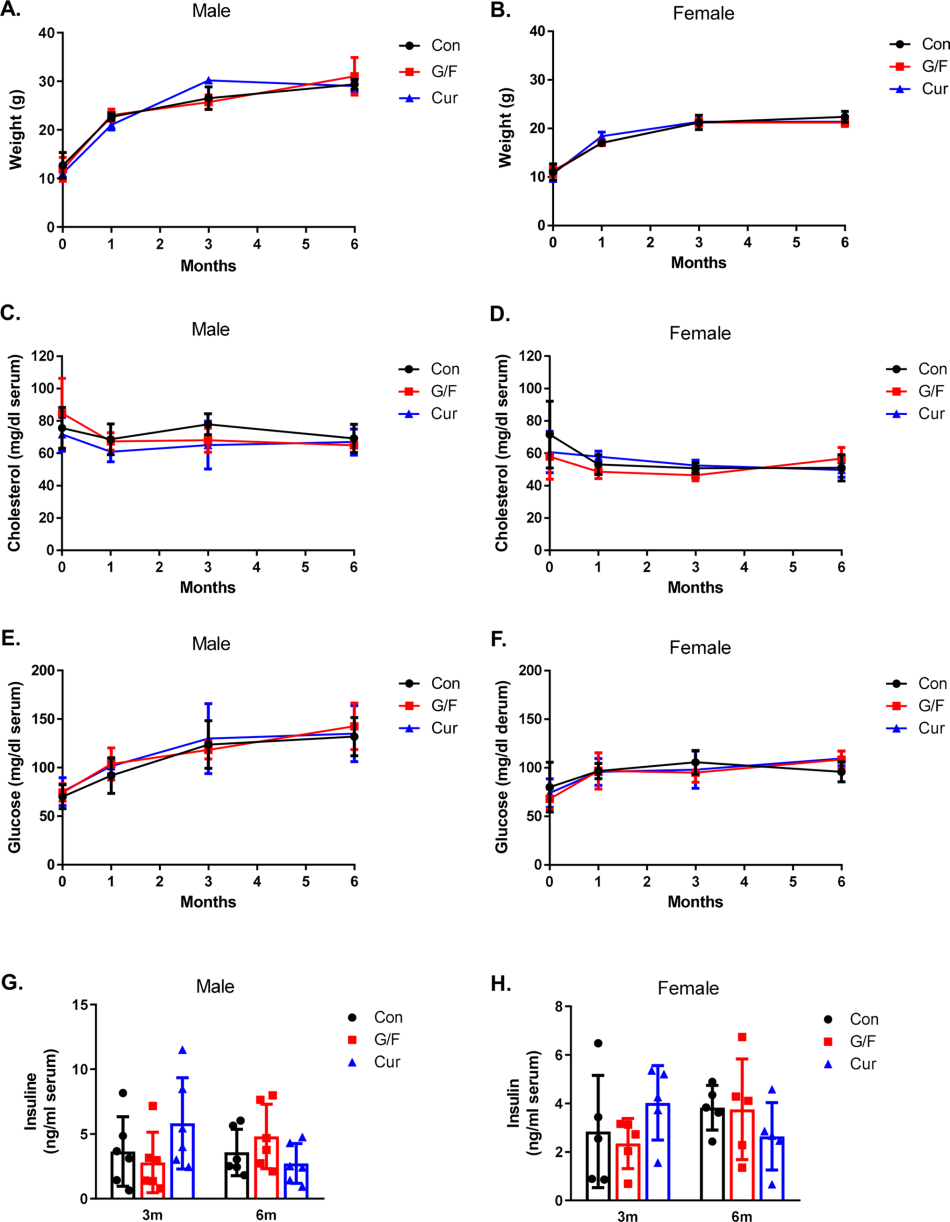
Body weight (**A**, **B**) and serum cholesterol (**C**, **D**), glucose (**E**, **F**), and insulin (**G**, **H**) concentration in 5xFAD mice fed with control diet, glucose/fructose-supplemented diet and currant-supplemented diet. Con: control diet, G/F: diet containing 1.75% (w/w) glucose/1.75% (w/w) fructose, Cur: diet containing 5% (w/w) currants paste. Data are expressed as mean ± SD (*n* = 6 male, 5 female-per condition)

Starting at 1 month of age, groups of male (*n* = 6) and female (*n* = 5) 5xFAD mice were exposed to either typical diet [control (Con) group] or diet containing 5% (w/w) Corinthian currants paste [currant (Cur) group] or sugar matched diet containing 1.75% (w/w) glucose/1.75% (w/w) fructose [(G/F) group] for 1, 3, and 6 months. At baseline and at the end of each diet intervention period, mice were weighted and blood was collected for biochemical measurements. During the experimental period, all of the animals gained weight, but there was no difference among groups of both sexes receiving the various dietary treatments (Fig. 1A, B). There was also no significant difference in serum cholesterol or glucose levels among the three groups at any time point of the treatment (Fig. 1C, D, E, F), as well as in serum insulin levels measured following 3 and 6 months of diet (Fig. 1G, H). Thus, Corinthian currant paste consumption does not seem to result in hyperglycemia or hyperlipidemia.

### Time-Dependent Effect of Currant Diet on Αβ42 Levels in the Brain of 5xFAD Mice

**Fig. 2:**
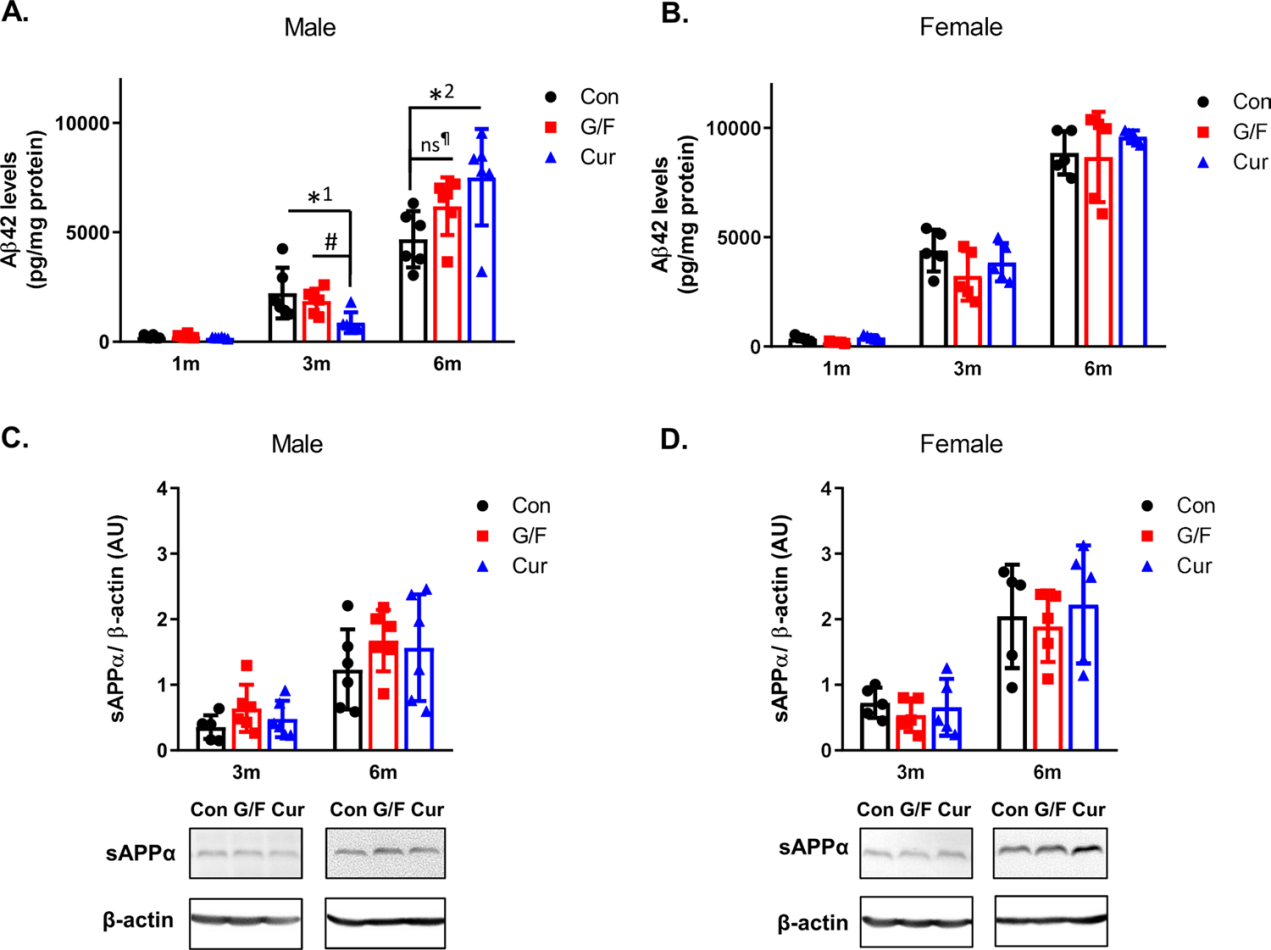
Levels of Aβ42 and sAPPα in 5xFAD mice fed with control diet, glucose/fructose-supplemented diet and currant-supplemented diet. **A**, **B** Aβ42 levels were measured in detergent-soluble cortex fractions by ELISA and normalized with respect to protein levels of fraction. **C**, **D** Levels of sAPPα and β-actin in soluble cortex fractions were measured following SDS-PAGE and Western blot. The normalized levels of sAPPα levels against β-actin levels are shown. Con: control diet, G/F: diet containing 1.75% (w/w) glucose/1.75% (w/w) fructose, Cur: diet containing 5% (w/w) currants paste. Data are expressed as mean ± SD (*n* = 6 male, 5 female-per condition). AU: arbitrary units. Statistical analysis for significance in differences between two groups per time point was performed using the unpaired two-tailed Student’s *t*-test. *p* values for statistically significant differences (*p* < 0.05) are shown. *^1^*p* = 0.024, *^2^*p* = 0.022, ^#^*p* = 0.008, ns: non-significant - ^¶^*p* = 0.072

To investigate the effect of currant diet on AD pathogenetic processes, we measured Aβ42 levels in the cortex of 5xFAD mice receiving currant-supplemented diet, glucose/fructose-supplemented diet and typical diet for 1, 3, and 6 months. The analysis showed that Aβ42 levels were similar in the three groups of male mice after 1 month of diet (Fig. 2A). Interestingly, after 3 months of diet, the male mice of currant group had lower Aβ42 levels as compared to male mice of control or G/F groups (Fig. 2A). However, following a longer intake of diet, for 6 months, currant group male mice had higher Aβ42 levels as compared to control male mice (Fig. 2A). Male mice of G/F group showed a trend towards increased Aβ42 levels as compared to control mice that did not reach though statistical significance. In contrast, no significant difference was observed in Aβ42 levels of female mice that received currant-supplemented diet as compared to mice that received the other two types of diet at any time point of the treatment (Fig. 2B). Analysis of the effect of currant-supplemented diet on the non-amyloidogenic pathway did not show any effect on sAPPα levels of male or female mice as compared to mice fed control and sugar-supplemented diets for 3 and 6 months (Fig. 2C, D). Overall, these findings indicate that currants intake has a time-dependent effect on Aβ42 quantity in the brain of male 5xFAD mice.

**Fig. 3:**
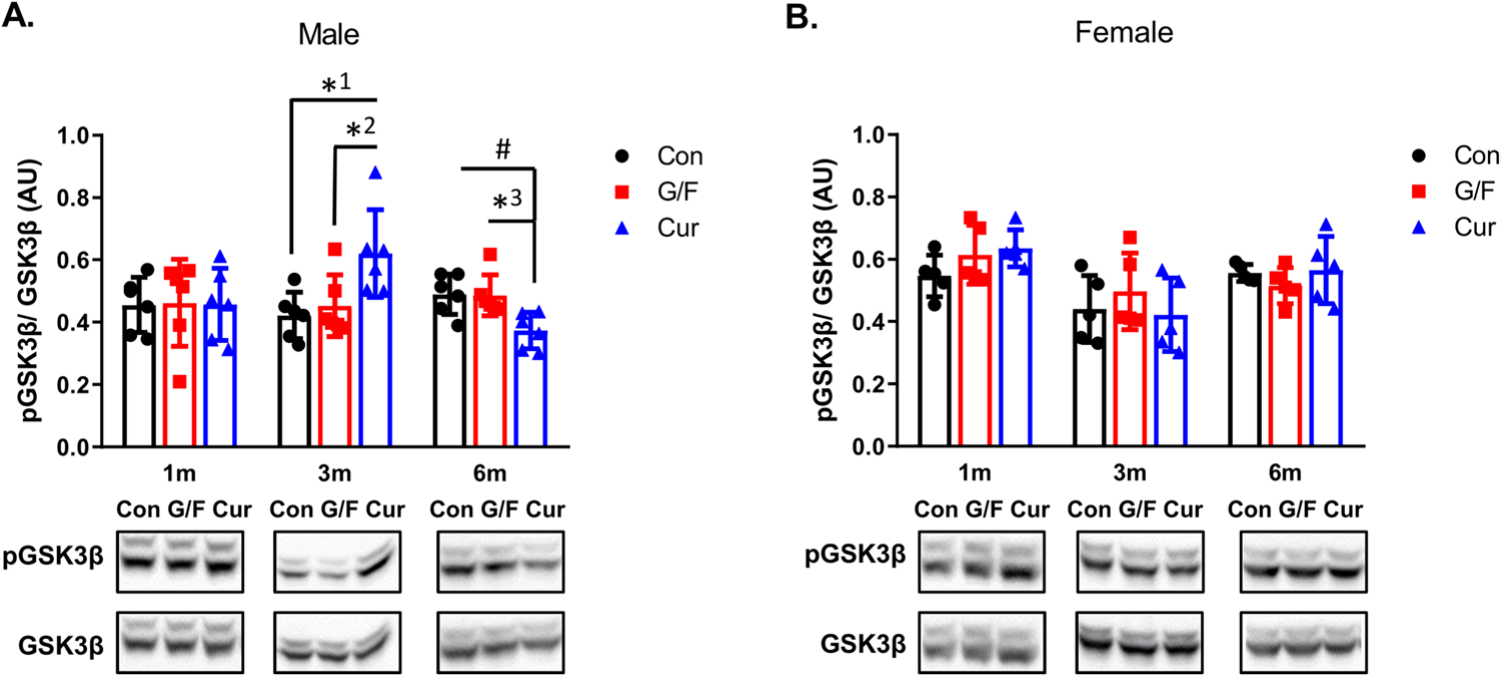
Phosphorylation of GSK3β in 5xFAD mice fed with control diet, glucose/fructose-supplemented diet, and currant-supplemented diet. Phospho-GSK-3-beta (pGSK3β) and total GSK3β levels in detergent-soluble cortex fractions were analyzed by SDS-PAGE and western blot. The normalized levels of pGSK3β against total GSK3β are shown. Con: control diet, G/F: diet containing 1.75% (w/w) glucose/ 1.75% (w/w) fructose, Cur: diet containing 5% (w/w) currants paste. Data are expressed as mean ± SD (*n* = 6 male, 5 female-per condition). AU: arbitrary units. Statistical analysis for significance in differences between two groups per time point was performed using the unpaired two-tailed Student’s *t*-test. *p* values for statistically significant differences (*p* < 0.05) are shown. *^1^*p* = 0.012, *^2^*p* = 0.035, *^3^*p* = 0.011, ^#^*p* = 0.008

Previous studies have suggested a possible cause-and-effect relationship between Aβ42 and glycogen synthase kinase 3β (GSK3β) activity, but the sequence of events is not known [36, 37]. We examined whether phosphorylation of GSK3β in Ser9 and thus inactivation of the enzyme is affected in 5xFAD mice fed the currant diet. As shown in Fig. 3A, 3-month currant male mice group displayed an increase in phosphorylation of GSK3β in the cortex, while 6-month currant male mice group displayed a decrease in phosphorylation of GSK3β, as compared to mice fed the other two types of diet. There was no difference in phosphorylation of GSK3β in currant female mice group as compared to mice that received the other two types of diet at any time point of the treatment (Fig. 3B). These findings show a similar pattern of changes in Aβ42 levels and pGSK3β/GSK3β levels in mice following the administration of different types of diet for 3 and 6 months.

**Fig. 4:**
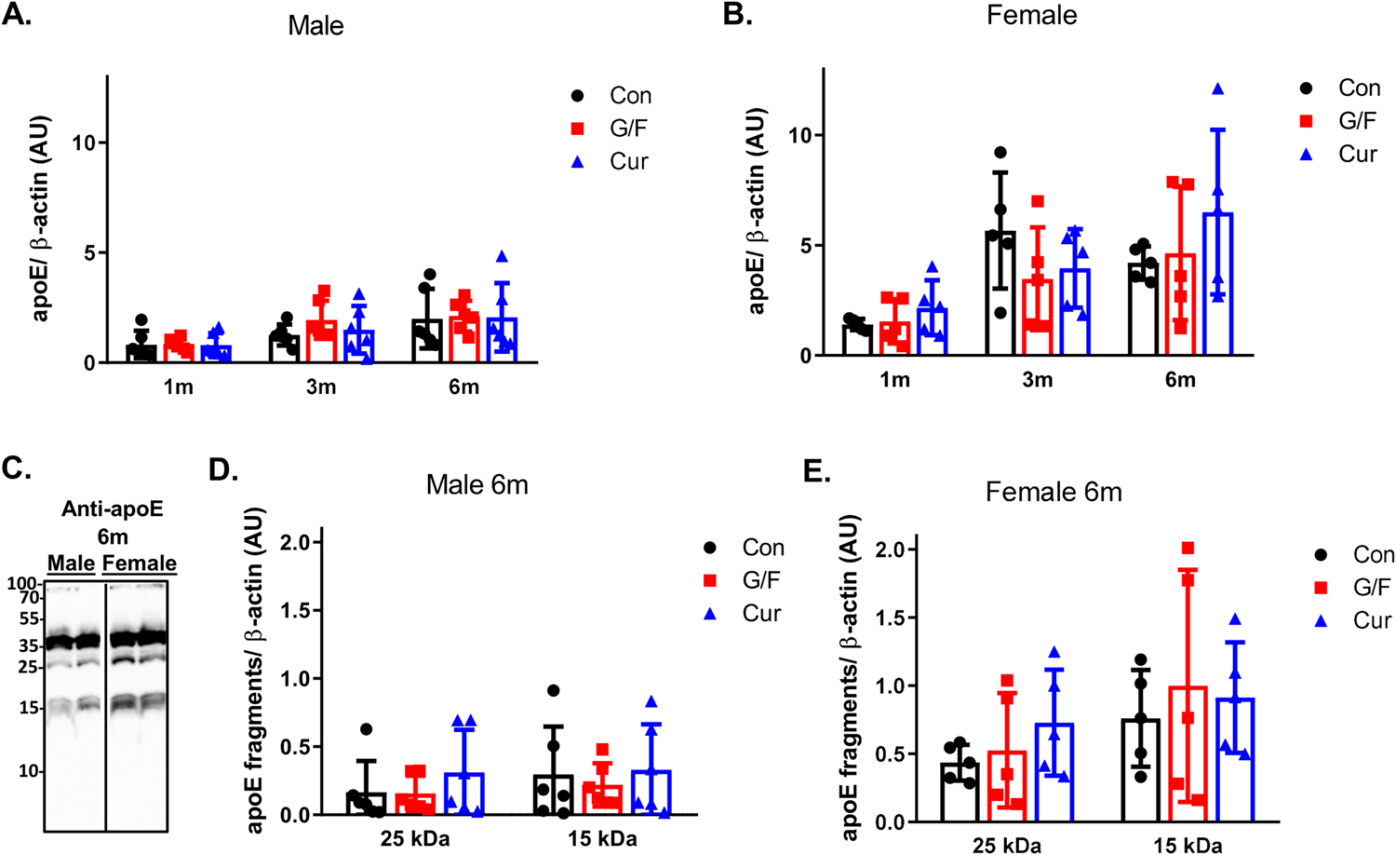
Levels of total apoE and apoE fragments in 5xFAD mice fed with control diet, glucose/fructose-supplemented diet, and currant-supplemented diet. Detergent-soluble cortex fractions were analyzed by SDS-PAGE and Western blot. Total apoE (**A**, **B**) and apoE fragments (**C**–**E**) levels were normalized to β-actin levels. ApoE fragments (**C**) are detectable in the brains of older 5xFAD mice (on diet for 6 months). Con: control diet, G/F: diet containing 1.75% (w/w) glucose/ 1.75% (w/w) fructose, Cur: diet containing 5% (w/w) currants paste. Data are expressed as mean ± SD (*n* = 6 male, 5 female-per condition). AU: arbitrary units

Since the amyloid pathology in 5xFAD mice has been linked to apoE fragmentation [38], we examined whether currant diet may have an effect on total apoE levels and apoE fragmentation in the cortex of 5xFAD mouse. Our analysis did not show any difference in total apoE levels among groups of either male or female mice receiving the various dietary treatments per time point (Fig. 4A, B). ApoE fragments with molecular weights of 25 and 15 kDa were detected only in older mice (on diet for 6 months) (Fig. 4C), as it was reported before [38]. There was no effect of currant diet on apoE fragmentation in the brain of mice (Fig. 4D, E).

**Fig. 5:**
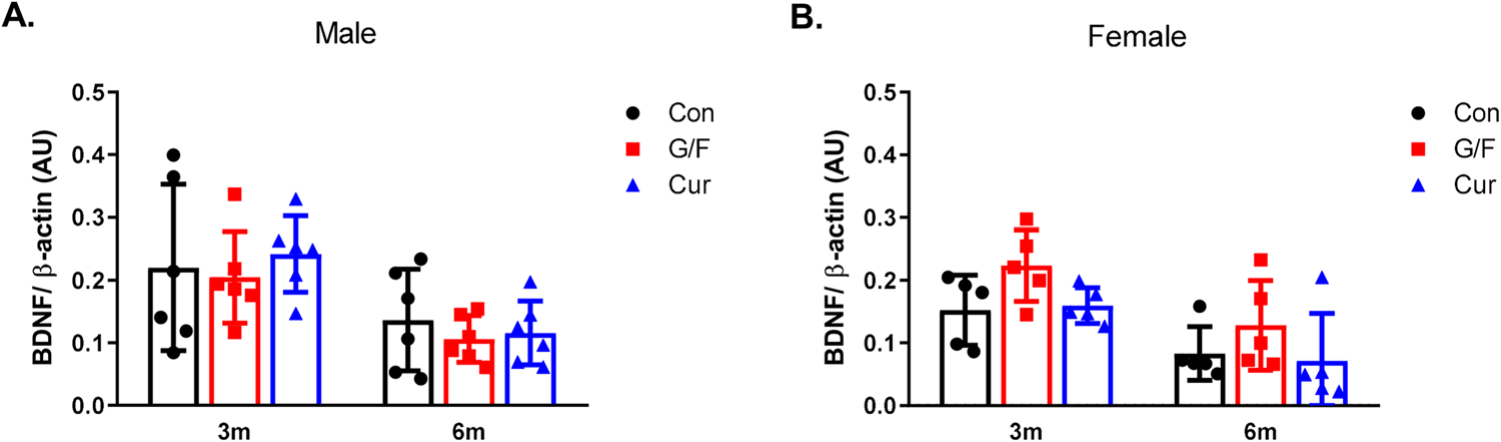
Levels of BDNF in 5xFAD mice fed with control diet, glucose/fructose-supplemented diet, and currant-supplemented diet. BDNF and β-actin levels in soluble cortex fractions were measured following SDS-PAGE and Western blot. BDNF levels were normalized to β-actin levels. Con: control diet, G/F: diet containing 1.75% (w/w) glucose/1.75% (w/w) fructose, Cur: diet containing 5% (w/w) currants paste. Data are expressed as mean ± SD (*n* = 6 male, 5 female-per condition). AU: arbitrary units

Brain-derived neurotrophic factor (BDNF) protein levels are dramatically reduced in the brains of AD patients and in normal brain tissue exposed to oligomeric Aβ [39, 40]. We examined whether the effect of currant diet on Aβ42 levels in male mice is associated with changes in cortical BDNF levels. As shown in Fig. 5, there was no effect in BDNF levels among groups of both sexes receiving the various dietary treatments for 3 or 6 months.

Collectively, these results show that currant-supplemented diet provided for a short period (3 months) in young male 5xFAD mice may have beneficial effects against AD progression by reducing brain Aβ42 levels. However, administration of currant-supplemented diet for a longer period (6 months) in male 5xFAD mice not only had no further beneficial effect but instead resulted in increase of brain Aβ42 levels.

### Time-Dependent Effect of Currant Diet on TNFα and IL-1β Levels in the Brain of 5xFAD Mice

**Fig. 6:**
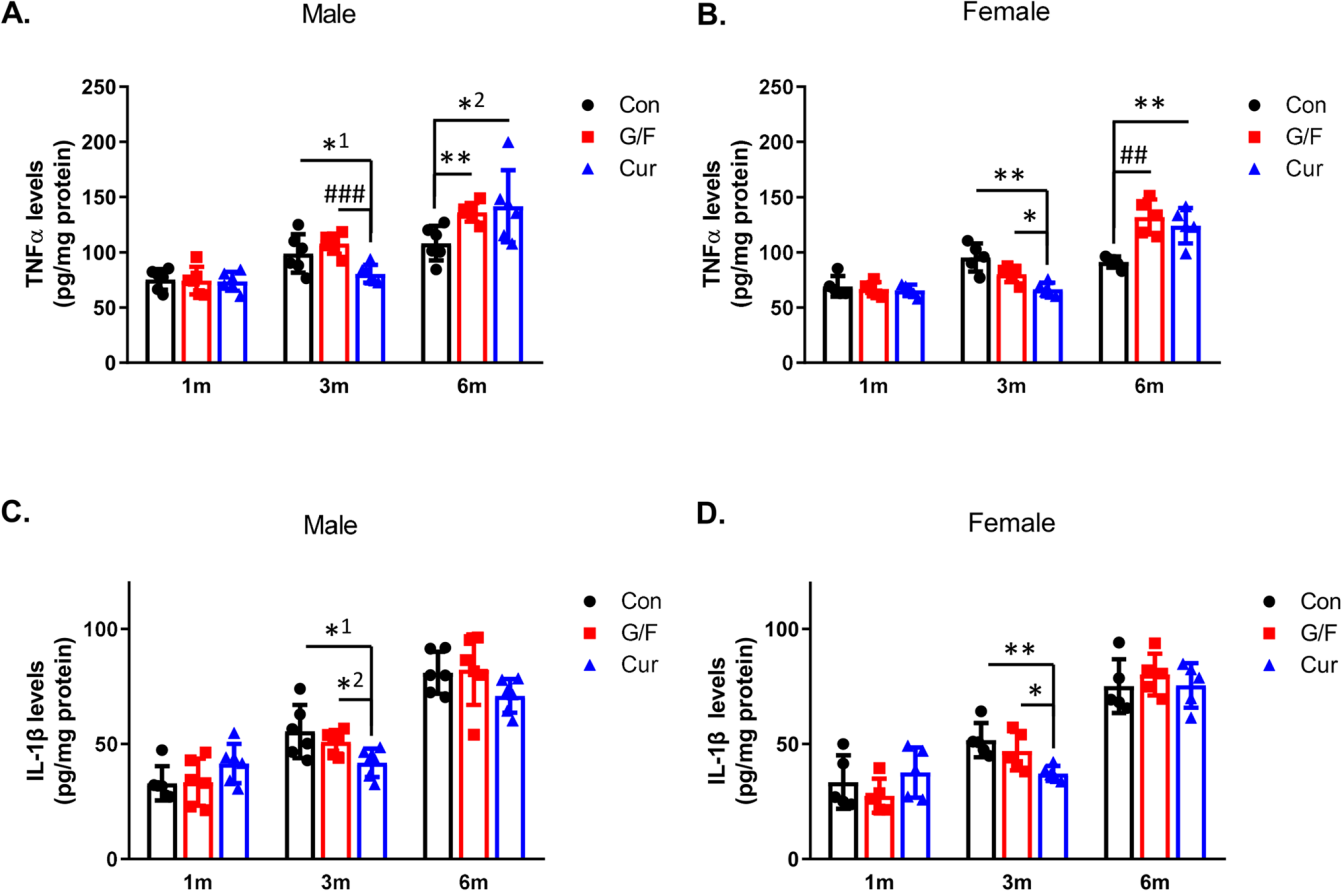
Levels of TNFα and IL-1β in 5xFAD mice fed with control diet, glucose/fructose-supplemented diet, and currant-supplemented diet. TNFα (**A**, **B**) and IL-1β (**C**, **D**) levels were measured in homogenated cortex fractions by ELISA and normalized with respect to protein levels of fraction. Con: control diet, G/F: diet containing 1.75% (w/w) glucose/ 1.75% (w/w) fructose, Cur: diet containing 5% (w/w) currants paste. Data are expressed as mean ± SD (*n* = 6 male, 5 female-per condition). Statistical analysis for significance in differences between two groups per time point was performed using the unpaired two-tailed Student’s *t*-test. *p* values for statistically significant differences (*p* < 0.05) are shown. **A** *^1^*p* = 0.041, *^2^*p* = 0.043, **p = 0.003, ^###^*p* = 0.0003. **B** *p = 0.012, **p = 0.002, ^##^*p* = 0.0006. **C** *^1^*p* = 0.028, *^2^*p* = 0.019. **D** *p = 0.042, **p = 0.004

In order to evaluate the effect of currant diet on neuroinflammation, another process that contributes to AD pathogenesis, we measured the levels of the key proinflammatory cytokines TNFα and IL-1β in the cortex of 5xFAD mice receiving currant-supplemented diet for 1, 3, and 6 months in comparison to normal diet and sugar-matched diet. After 1 month of diet, TNFα and IL-1β levels were similar in the three groups of male and female mice (Fig. 6A, B, C, D), while 3-month currant group mice of both sexes had lower levels of both TNFα and IL-1β, as compared to control and G/F groups (Fig. 6A, B, C, D). However, after 6 months, both male and female 5xFAD mice that received currant-supplemented diet had higher TNFα levels as compared to mice that received normal diet (Fig. 6A, B). Similar high TNFα levels to those of mice on currant diet had the mice that received sugar-supplemented diet (Fig. 6A, B). Currant diet administered for 6 months also had no beneficial effect on IL-1β levels. Male and female mice that received currant-supplemented diet, sugar-matched diet, and normal diet had similarly increased IL-1β levels (Fig. 6C, D).

**Fig. 7:**
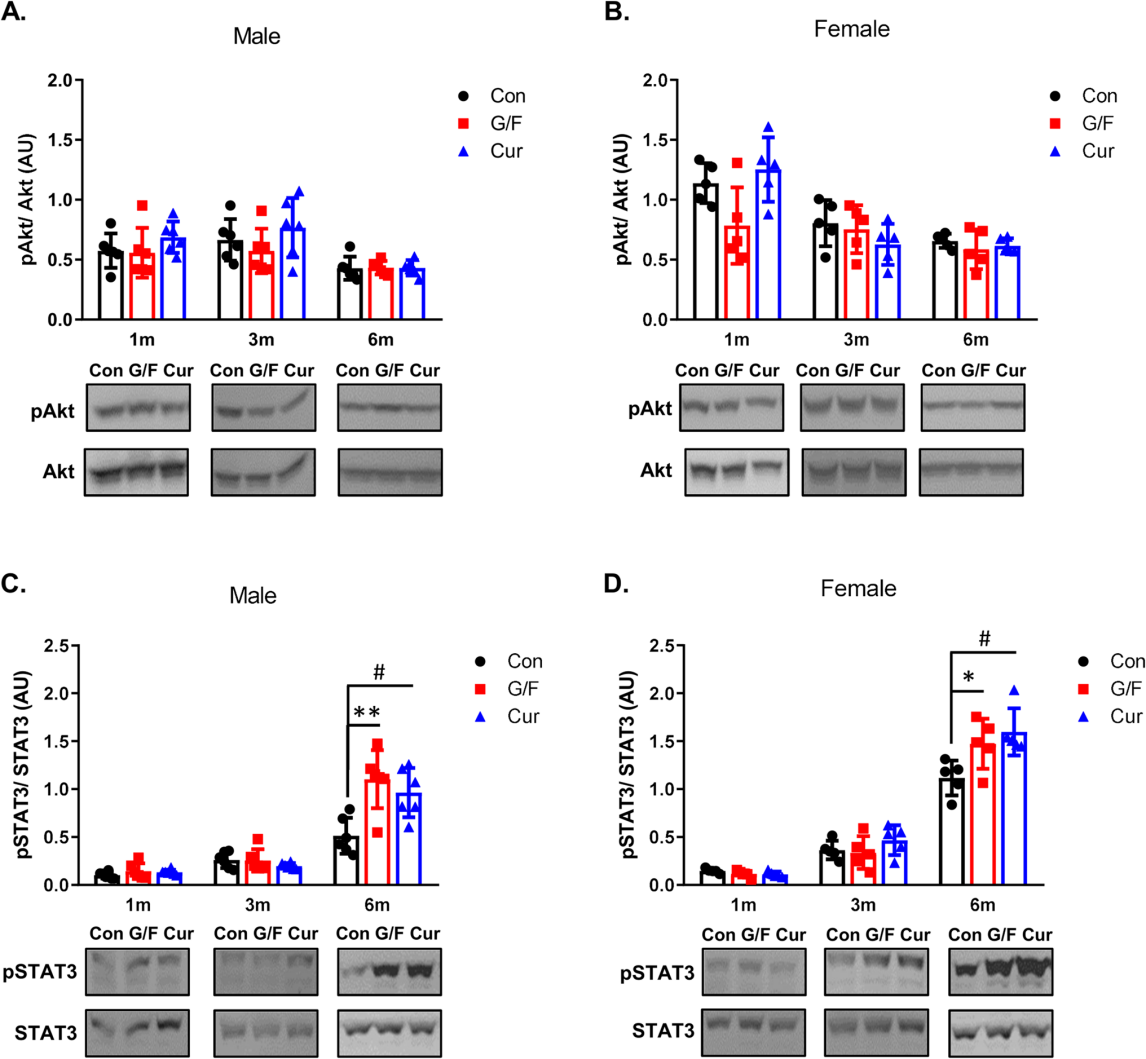
Phosphorylation of Akt and STAT3 in 5xFAD mice fed with control diet, glucose/fructose-supplemented diet, and currant-supplemented diet. Phospho-Akt (pAkt) and total Akt (**A**, **B**), as well as phospho-STAT3 (pSTAT3) and total STAT3 (**C**, **D**) levels in detergent-soluble cortex fractions were analyzed by SDS-PAGE and Western blot. The normalized levels of pAkt against total Akt and pSTAT3 against total STAT3 are shown. Con: control diet, G/F: diet containing 1.75% (w/w) glucose/ 1.75% (w/w) fructose, Cur: diet containing 5% (w/w) currants paste. Data are expressed as mean ± SD (*n* = 6 male, 5 female-per condition). AU: arbitrary units. Statistical analysis for significance in differences between two groups per time point was performed using the unpaired two-tailed Student’s *t*-test. *p* values for statistically significant differences (*p* < 0.05) are shown. **C** ^#^*p* = 0.006, **p = 0.002. **D** *p = 0.038, ^#^*p* = 0.008

Neuroinflammation is associated with signaling pathways via a crosstalk of cytokines with signal transducers and activators of transcription pathways [41]. To probe for a mechanism behind the changes in TNFα and IL-1β levels, we examined the effect of currant- or sugar-supplemented diet on Akt and STAT3 pathways. Activated PI3K/Akt and STAT3 pathways have been suggested to contribute to neurodegenerative processes [41–43] and are targets of synthetic and natural compounds for the prevention or treatment of AD [44, 45]. Our analysis did not show any difference in phosphorylation of Akt in the cortex of mice receiving the various dietary treatments per time point (Fig. 7A, B). The phosphorylation of STAT3 in the cortex was also not affected by the type of diet administered in mice for 1 and 3 months, but there was increase in phosphorylation of STAT3 in male and female mice on currant- or sugar-supplemented diet for 6 months as compared to mice on normal diet (Fig. 7C, D). The increase in phosphorylation of STAT3 could be related to the increase of TNFα secretion in the cortex of both male and female mice that received currant- or sugar-supplemented diet for 6 months.

### Time-Dependent Decrease of Hippocampal TNFα Expression in 5xFAD Mice on Currant-Supplemented Diet

**Fig. 8:**
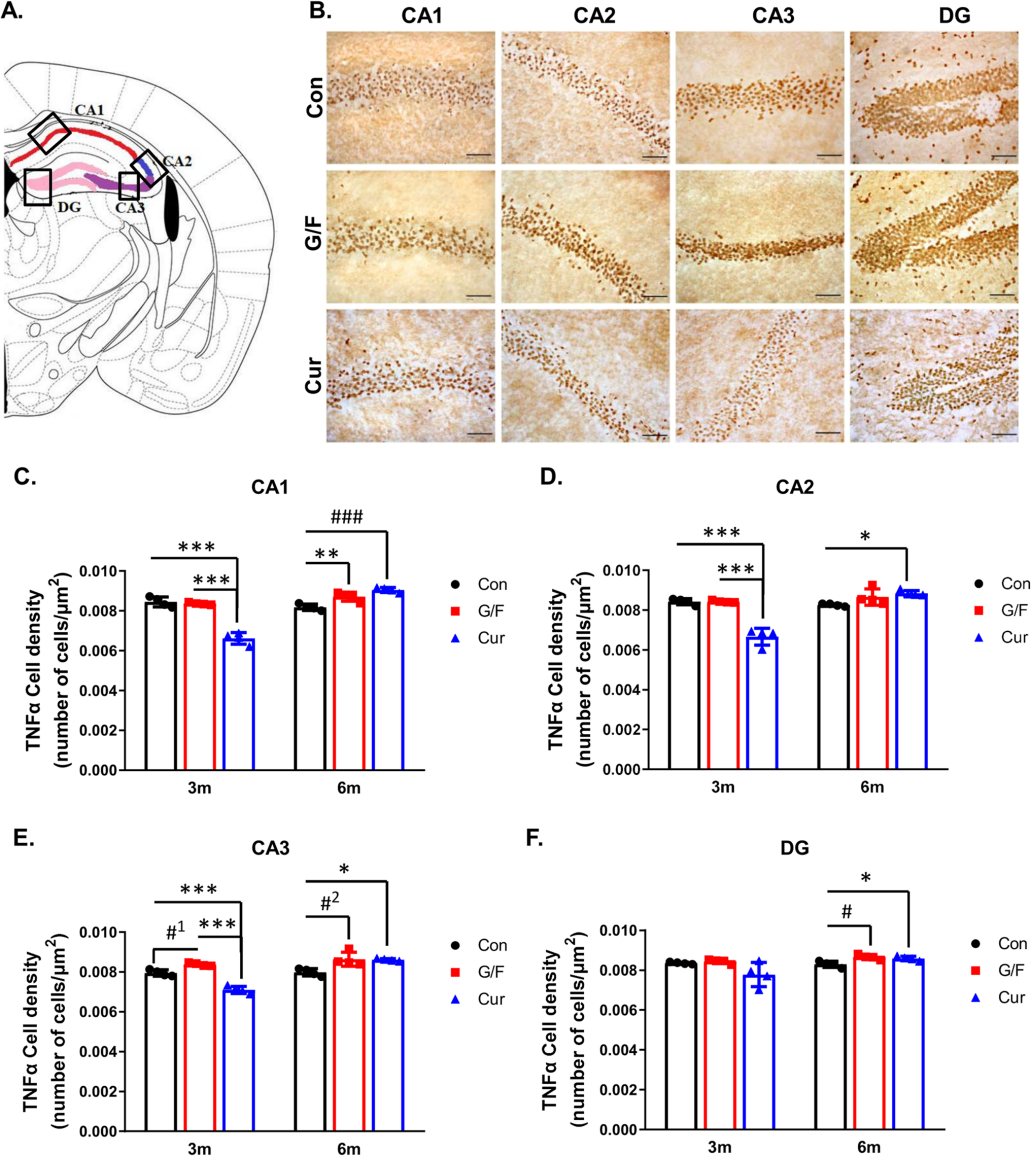
The effects of currant- and sugar-supplemented diet conditions on hippocampal TNFα immunoreactivity in 5xFAD male mice. **A** Drawing indicates the coronal level of hippocampus (Paxinos and Franklin mouse brain atlas, 2013), showing the CA1, CA2, CA3, and DG subdivisions. **B** Representative photomicrographs of TNFα immunoreactivity in hippocampal subdivisions in all experimental groups following a 3-month diet. Scale bar = 0.05 mm. **C**–**F** TNFα immunoreactivity in hippocampal subdivisions of mice following administration of different diet types for 3 and 6 months. Con: control diet, G/F: diet containing 1.75% (w/w) glucose/ 1.75% (w/w) fructose, Cur: diet containing 5% (w/w) currants paste. Data are expressed as mean ± SD (*n* = 4 per condition). Statistical comparisons were performed using a two-way analysis of variance (ANOVA) followed by Post-hoc Tukey comparisons with independent variable the “type of diet”. *p* values for statistically significant differences (*p* < 0.05) are shown. **C** **p = 0.004, ^###^*p* = 0.0002, ***p < 0.0001. **D** *p = 0.024, ***p < 0.0001. **E** *p = 0.010, ^#1^*p* = 0.006, ^#2^*p* = 0.008, ***p < 0.0001. **F** *p = 0.028, ^#^*p* = 0.008

Following the analysis of the effect of currant- and sugar-supplemented diet on TNFα levels in the cortex of 5xFAD mice, we also examined the effect of diet on TNFα expression in the hippocampus of mice. We analyzed male mice that, in addition to the reduction of cortical TNFα levels, showed a reduction in Aβ42 levels following a 3-month currant-supplemented diet. Administration of three types of diets in 5xFAD mice for 3 and 6 months resulted in alterations of TNFα^+^ cell immunoreactivity in hippocampal subdivisions (Fig. 8A, B, C, D, E, F). Two-way ANOVA indicated significant simple main effects (time, diet condition) and interaction between the two experimental factors (time × diet condition) in all subdivisions of the hippocampus studied (Table 1). Specifically, the 3-month currant group showed a decrease of TNFα cell immunodensity in CA1 hippocampal layer compared to control and G/F groups (Fig. 8C). In contrast, 6-month currant group as well as 6-month G/F group showed increased TNFα^+^ cells compared to the control group (Fig. 8C). Similarly, in CA2 layer, a 3-month currant supplemented-diet reduced TNFα + cells as compared to control and G/F groups, while 6-month currant-enriched diet resulted in a small but significant increase of TNFα^+^ cell density (Fig. 8D). Likewise, TNFα^+^ cell immunoreactivity in CA3 hippocampal layer was significantly decreased following 3-month currant-supplemented diet compared to control and G/F groups, while TNFα increased after 6 months of both currant- and sugar-supplemented diet compared to control group (Fig. 8E). In addition, TNFα^+^ cells were also increased in the G/F group after 3 months of diet in comparison to control group (Fig. 8E). In Dentate Gyrus (DG) all 3-month diet conditions had no effect on TNFα cell immunoreactivity (Fig. 8F). However, 6 months of diet led to increases on TNFα cell immunoreactivity in the currant and G/F groups compared to the control group (Fig. 8F). Overall, our analysis shows that currant supplemented-diet after 3 months decreased TNFα immunoreactivity in the majority of hippocampal subdivisions, while after 6 months of currant diet the density of TNFα expressing cells was increased. Sugar-supplemented diet also increased TNFα^+^ cell density in the majority of hippocampal subdivisions after 6 months.

**Table 1:** Two-way ANOVA statistical analyses of TNFα cell immunoreactivity in 5xFAD male mice (n = 4) hippocampal layers/sub-regions

Hippocampus		TNFα	
		F_1,23_	p
CA1	Time	103.39	*p* < 0.001
	Diet condition	25.324	*p* < 0.001
	Time × Diet condition	97.848	*p* < 0.001
CA2	Time	50.555	*p* < 0.001
	Diet condition	20.163	*p* < 0.001
	Time × Diet condition	47.388	*p* < 0.001
CA3	Time	57.669	*p* < 0.001
	Diet condition	25.957	*p* < 0.001
	Time × Diet condition	33.539	*p* < 0.001
DG	Time	8.845	*p* = 0.008
	Diet condition	3.809	*p* = 0.042
	Time × Diet condition	5.474	*p* = 0.014

**Fig. 9:**
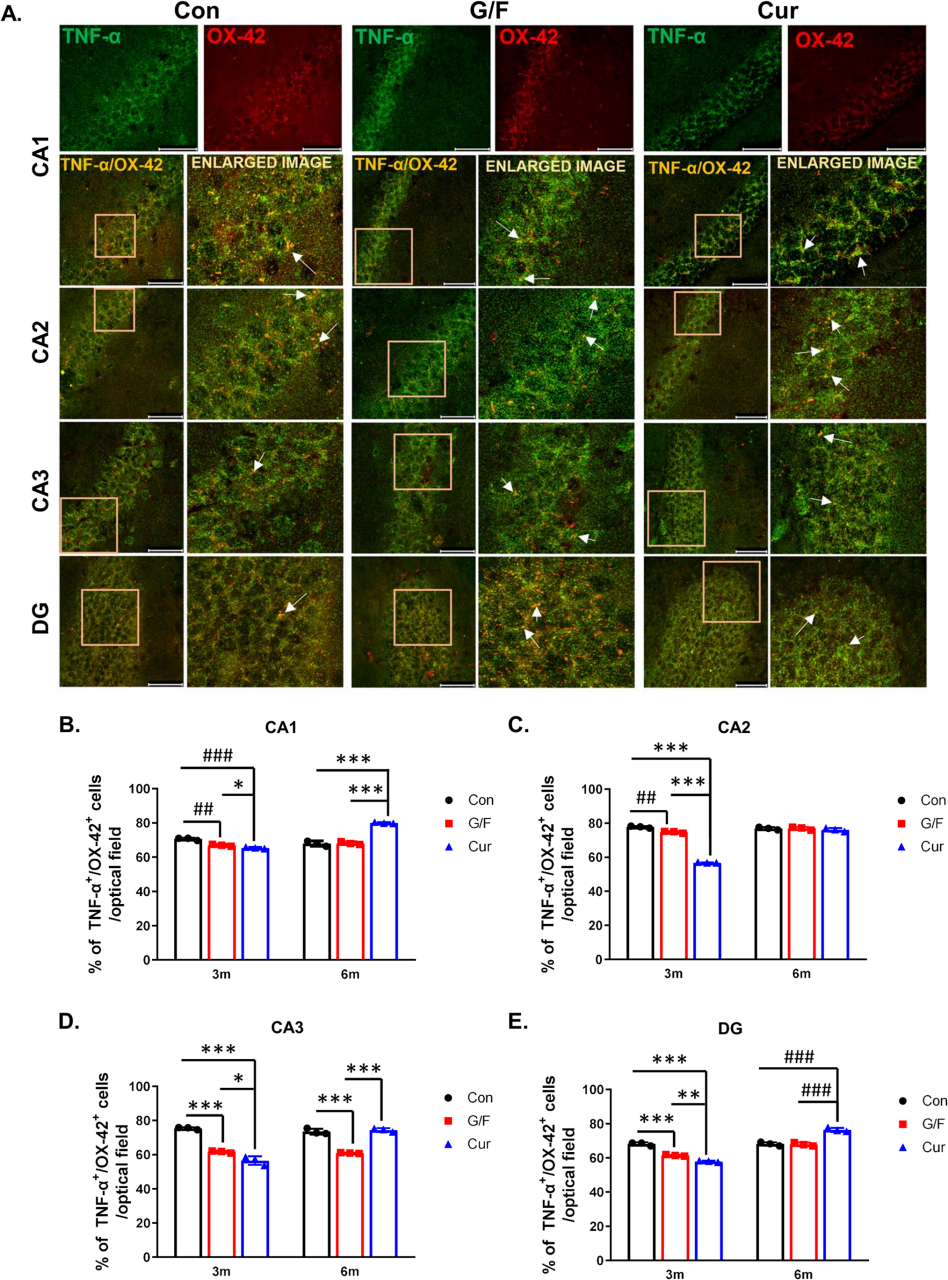
Double immunofluorescence labeling for TNFα and microglial marker OX-42 (CD11b) in the hippocampus of 5xFAD male mice following control, G/F or currant diet conditions for 3 and 6 months. **A** Representative photomicrographs showing the colocalization of TNFα-positive cells (green) with the marker of activated microglia CD11b (clone OX-42 (red)) in control and treated animals after 6 months of diet in CA1, CA2, CA3, and DG hippocampal subdivisions. Fields enclosed in boxes are shown at higher magnification to the right. The arrows indicate double-labelled cells. Scale bar = 0.05 mm. **B**–**Ε** Percentage of TNF-α + /CD11b + cells in hippocampal subdivisions of mice following administration of the three diet types for 3 and 6 months. Con: control diet, G/F: diet containing 1.75% (w/w) glucose/ 1.75% (w/w) fructose, Cur: diet containing 5% (w/w) currants paste. Data are expressed as mean ± SD (*n* = 3 per condition). Statistical comparisons were performed using a two-way analysis of variance (ANOVA) followed by Post-hoc Tukey comparisons with independent variable the “type of diet.” *p* values for statistically significant differences (*p* < 0.05) are shown. **B** *p = 0.048, ^##^*p* = 0.0008, ^###^*p* = 0.0001, ***p < 0.0001. **C** ^##^*p* = 0.0008, ***p < 0.0001. **D** *p = 0.016, ***p < 0.0001. **E** **p = 0.002, ^###^*p* = 0.0002, ***p < 0.0001

Double immunofluorescence experiments, at the different diet conditions of 5xFAD mice, showed that TNFα is co-expressed in all cases and hippocampal layers with the CD11b integrin (clone OX-42), supporting the TNFα expression in activated microglial cells (Fig. 9A and Supplemental Fig. 1). However, we cannot exclude the possibility that a small percentage of CD11b^+^ cells represents vascular associated macrophages [46] known to have a role in regulation of vascular amyloid-beta plaques deposition [47]. Importantly, TNFα^+^ cells co-expressing the CD11b (OX-42) marker represented the vast majority, ranging from 68 to 78%, at all hippocampal layers, in control mice, and at both time points studied. The percentage of TNFα^+^/CD11b^+^ (OX-42) was found reduced (ranging from 61 to 74%) in mice following a 3-month glucose/fructose-supplemented diet compared to control mice and even less (56% to 65%) in mice that received a 3-month currant supplemented-diet as compared to control and G/F groups (Fig. 9B, C, D, E). However, following a 6-month diet, the percentage of TNFα^+^ cells that were also CD11b positive in the currant group were increased (74 to 80%) at similar or higher values of those of the control group (Fig. 9B, C, D, E). Similarly high was the percentage of TNFα + /CD11b^+^ (OX-42) at the majority of hippocampal layers in mice that received the sugar supplemented-diet for 6 months (Fig. 9B, C, D, E). Overall, these results are in accordance with our previous findings (Fig. 6, Fig. 8) on the time-dependent effect of currant-supplemented diet on TNFα expression in 5xFAD mice and highlight the involvement of microglial activation in the observed changes of TNFα levels.

### The Effect of Currant Phenolic Extract on TNFα Secretion from BV2 Microglial Cells

**Fig. 10:**
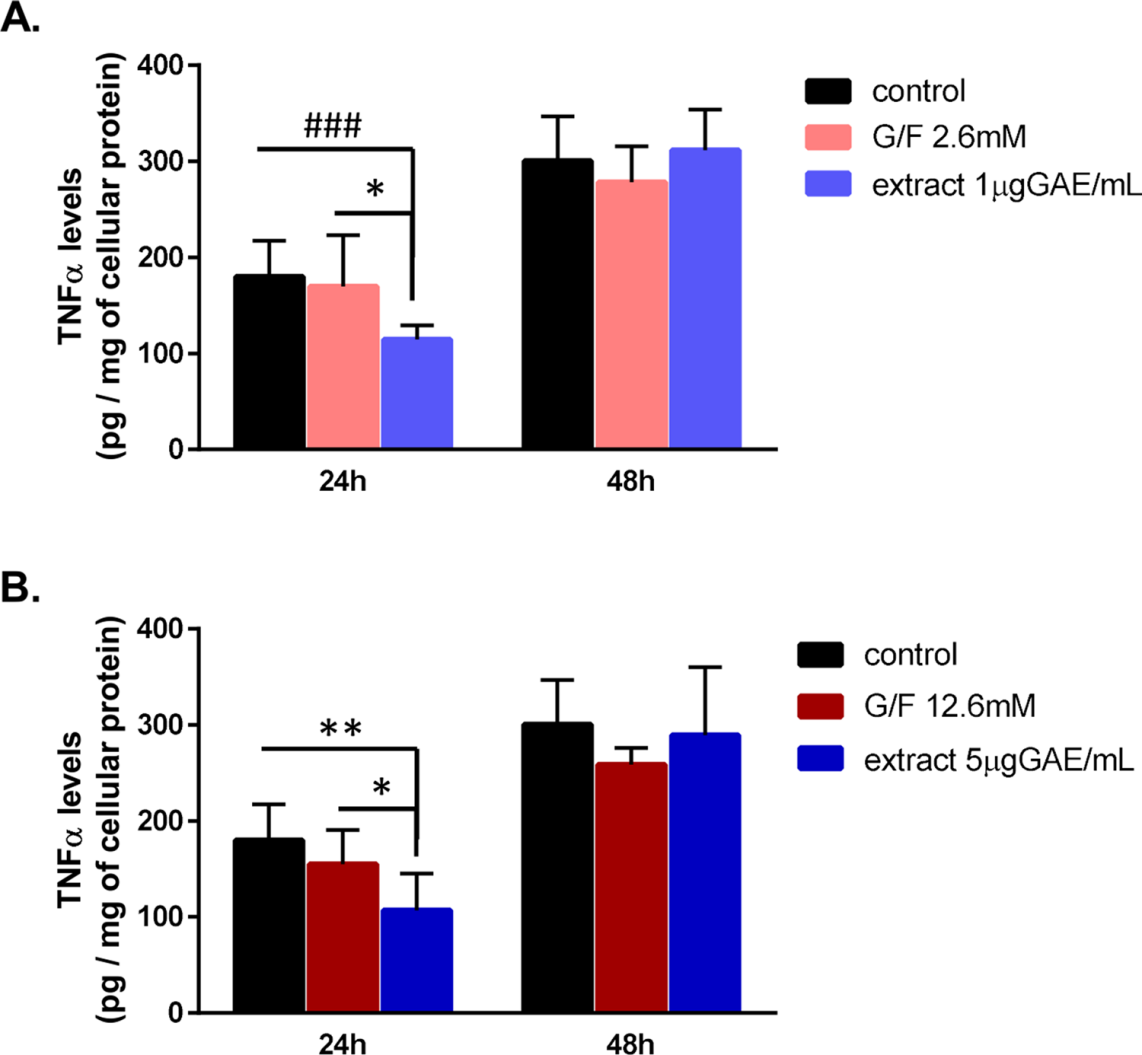
The effect of currant phenolic extract in TNFα secretion from BV2 microglial cells. BV2 cells were incubated in the absence (control) or presence of currant polar phenolic extract at concentrations of 1 μg GAE/mL (**A**) and 5 μg GAE/mL (**B**) or glucose/fructose (G/F, 1:1 mol/mol) mixture at concentrations of 2.6 mM (**A**) and 12.6 mM (**B**) that match the glucose/fructose concentration of currant extract. TNFα levels were measured in cell medium by ELISA and normalized with respect to cellular protein levels. Data are expressed as mean ± SD from three independent experiments performed in duplicate. Statistical analysis for significance in differences between two groups per time point was performed using the unpaired two-tailed Student’s *t*-test. *p* values for statistically significant differences (*p* < 0.05) are shown. **Α** *p = 0.014, ^###^*p* = 0.0004. **B** *p = 0.026, **p = 0.0018

Several effects of raisins and currants have been ascribed to their phenolic components and raisin/currants phenolic extracts have been shown to reduce cellular inflammatory responses [18, 19]. Thus, we wanted to examine whether the currant phenolic extract could affect TNFα secretion from brain cells as compared to a sugar-matched solution. Given the involvement of microglia in neuroinflammation in AD [41], as well as in our mice studies, we incubated BV2 microglial cells in the absence or presence of a currant polar phenolic extract as well as of a solution of glucose/fructose, that matches the glucose/fructose concentration of currant extract, for 24 and 48 h. The currant polar phenolic extract was used at two concentrations (expressed as µg of GAE per ml), shown previously to restore the viability of neuronal cells in the presence of apoE4 pathogenic forms and to prevent changes in cellular redox status [15]. There were no statistically significant changes in cell proliferation among cells treated in the absence or presence of currant phenolic extract or glucose/fructose solution for each time point (Supplemental Fig. 2). Treatment of cells with the currant phenolic extract at concentrations of 1 and 5 µg GAE/ml for 24 h reduced the levels of secreted TNFα as compared to untreated cells or sugar-treated cells (Fig. 10A, B). However, treatment of cells with the currant phenolic extract for a longer period, 48 h, increased secreted TNFα to levels that are similar to those of untreated cells or sugar-treated cells (Fig. 10A, B). Overall, these findings show a short-term decrease of TNFα secretion from microglial cells treated in the presence of currant phenolic extract that is diminished following a longer treatment.

## Discussion

Our findings illustrate that a 3-month currant-supplemented diet administered in young 5xFAD mice (1-month-old) results in benefits on AD-related pathology (reduction in Aβ42 and inflammatory cytokine levels), as compared to sugar-matched or control diet. However, a 6-month currant-supplemented diet has no beneficial effect in 5xFAD mice as compared to the other two types of diets. Furthermore, especially for brain TNFα levels, it was observed that both the currant and sugar-matched diets administered for 6 months result in even more pronounced increase of TNFα levels compared to control diet. These findings suggest that additional dietary Corinthian currant may be useful to reduce brain Aβ42 levels and microglial activation, thereby attenuating neuroinflammation at the early stages of the disease, but at later stages of disease when AD pathology has imposed a heavy and irreversible burden in the brain no beneficial effects are observed. Furthermore, our studies show that a long-term intake of a glucose/fructose-containing diet such as currant-supplemented diet, even if it has a low glycemic index and despite the lack of any effect in serum glucose levels, can result on detrimental effects regarding neuroinflammation. To the best of our knowledge, the effect of chronic intake of low levels of sugars from unprocessed natural products on brain health has not been reported before.

Glycemic index values for raisins have been found to be low in several groups such as healthy adults, healthy sedentary individuals and prediabetic adults [48, 49]. A randomized crossover study carried out with healthy and diabetic subjects that received 74 g of Corinthian currants or 50 g of glucose as a reference food showed that currants intake reduced postprandial glucose and insulin responses, both in healthy and diabetic subjects [50]. A 12-week randomized controlled trial, in which subjects with modestly elevated blood glucose levels consumed dark raisins (90 kcal/serving) three times daily as compared to processed snacks, showed that regular consumption of currants may reduce glycemia and cardiovascular risk factors [51]. Overall, these studies indicated that currants are a beneficial dietary choice both for diabetics and healthy individuals and have no detrimental effect on glycemic and insulinemic responses. No interventional study with currants or other varieties of raisins has been performed though in AD patients. Our analyses in the AD mouse model 5xFAD showed no effect of currant diet on serum glucose and insulin levels for any time point as compared to sugar-matched or control diet. Furthermore, currant diet had no effect on mouse body weight or cholesterol levels. Therefore, 5xFAD mice do not display any hyperphagic response to sweet diet, altered glycemia or hypercholesterolemia that could account for the observed changes in Aβ levels and neuroinflammation.

5xFAD mice generate Aβ42 almost exclusively and rapidly accumulate massive cerebral Aβ42 levels. At 1–2 months of age, Aβ42 is found mainly within neurons, while extracellular amyloid deposition (and gliosis) begins at 2 months and reaches quickly a very large burden [28, 29]. Female 5xFAD mice display earlier onset of Aβ pathology than their male counterparts [52]. The sex differences in Aβ42 levels are accompanied in our study by sex differences in the effect of 3- and 6-months currant and sugar-matched diet in Aβ42 levels. The reduction in Aβ42 levels that is found only in male, but not in female, mice following currant treatment for 3 months could be attributed to the lower burden of Aβ42 in the brain of male mice at this age as compared to age-matched female mice. Furthermore, it could be possible that the much higher Aβ42 levels in female as compared to male mice after 6 months of diet do not allow any observation of diet-dependent changes in Aβ42 levels in these mice as it is observed in age-matched male mice. In other transgenic AD mouse models (e.g., APP751SL, APP/PSEN1, 3xTg) the female animals also display increased brain Aβ burden, while several studies have shown a greater prevalence of AD in women as well [53]. The sex differences in AD have been linked to a variety of factors, including genetics, functional and structural brain changes, as well as sex-related differences in various comorbidities (e.g., diabetes, hypertension, atherosclerosis), but the exact mechanisms remain to be unraveled [53, 54]. Future analyses of the longitudinal and sex-dependent effects of Corinthian currant consumption compared to a sugar-matched diet in appropriate animal models, that include combinations of different conditions reflecting comorbidities in AD, and humans could possibly provide useful information for the understanding and prevention of AD.

The pattern of changes in Aβ42 levels in mice following the administration of different types of diet for 3 and 6 months follows the pattern of changes in pGSK3β/GSK3β levels. Previous studies have suggested a possible cause-and-effect relationship between Aβ42 and GSK3β activity, but the sequence of events is not known. It has been shown that inhibition of GSK3β enables the clearance of Aβ in 5xFAD mice [36]. Thus, it could be possible that the effect of a currant-enriched diet on GSK3β activity via phosphorylation might be responsible for the observed changes in Aβ42 levels in the brain of male mice after 3 and 6 months on that diet. However, the different types of diet have no effect on pGSK3β/GSK3β levels in female mice, indicating that the currant-enriched diet could affect Aβ42 levels in male mice by other, non-related to GSK3β activity, mechanisms. In addition, it has been proposed that Aβ could affect GSK3β activation since treatment of cortical slices with oligomeric Aβ42 forms decreases the phosphorylation of GSK3β without any effect on total GSK3β levels [37]. Thus, the effects of currant-enriched diet on Aβ42 levels could have an impact on the observed changes in pGSK3β/GSK3β levels in the brain of male mice after 3 and 6 months.

TNFα and IL-1β levels in 5xFAD mice brains did not display substantial sex-dependent differences, in accordance with a previous study that also showed lack of substantial sex-dependent differences for the mRNA expression levels of these cytokines [55]. Therefore, it is reasonable that male and female mice displayed similar diet-dependent changes on TNFα and IL-1β levels in our study.

The increase of brain pro-inflammatory cytokine levels in AD has been proposed to contribute to disease progression [4]. Certain cytokines, such as TNFα, IL-1β, IFN-γ, and IL-6, dependently or independently of Aβ have been shown to contribute to brain cell death [56]. Impaired brain glucose metabolism has been proposed to be intrinsic to AD pathogenesis and could induce inflammatory responses in AD brain and exacerbate the disease pathology [57, 58]. Previous studies have associated diabetes (both type-1 and type-2) with AD, implicating hyperglycemia and abnormal insulin signaling in AD. However, since glucose transport from the peripheral circulation across the blood–brain barrier into the brain and then to neurons is tightly regulated and largely independent of insulin [59, 60], it is not clear whether abnormalities of brain glucose homeostasis in AD are related to peripheral glucose concentration. A recent study showed association between higher concentrations of plasma fasting glucose measured before death, as well as of greater increases in fasting plasma glucose over time, with higher brain tissue glucose concentrations in AD patients, but these associations were not driven by individuals with diabetes [58]. The average follow-up interval for measurements of plasma glucose was 19 years, and the average interval between the last available fasting plasma glucose measurement and death was 5 years [58]. Our findings show that, despite no effect in fasting plasma glucose or insulin levels, long-term administration of sugar-containing diets, such as the currant-supplemented and glucose/fructose-matched diets, can enhance neuroinflammation in the 5xFAD AD mouse model. These findings, could indicate that the sugar content of currants may have a role in the long-term enhancement of neuroinflammation by the currant diet. Further studies are necessary to examine whether longer administration of sugar-containing diets in aging 5xFAD mice would result in changes in serum glucose or insulin levels and whether long-term administration of currant-supplemented diet can affect neuroinflammation in non-AD mice (C57BL/6 mice) in comparison to normal diet and glucose/fructose-containing diet.

The effects of hyperglycemia have been extensively studied, showing that it can promote microglial numbers expansion and astrocytosis in the hippocampus and peripheral recruitment of leukocytes to the cerebral vasculature [61], while high glucose can enhance inflammatory cytokine secretion by stimulated human astrocytes [62]. However, the effect of chronic treatment of brain cells with low glucose levels is largely unexplored. Continuous supply of glucose from the circulation to the brain is critical to fulfill many essential functions, including ATP generation, oxidative stress management, and synthesis of neurotransmitters, neuromodulators, and structural components [63, 64]. Whether, chronic availability of low glucose levels in plasma leads to disruption of glucose delivery and metabolism in the brain, affecting nerve cell survival and impairing brain function needs further investigation.

A recent study has suggested that another mechanism driving AD pathogenesis could be impaired cerebral fructose metabolism [65]. Fructose consumption, but at much higher amounts (15%) than those used in our study (1.75%), has been shown to reduce hippocampal synaptic plasticity underlying cognitive performance in C57BL/6 male mice [66]. Furthermore, treatment of rats with 15% fructose induced alterations of the organization of genes in brain region-specific networks interrelating cell metabolism, immune function, inflammation, and cell communication [67]. Whether long-term low concentrations of dietary fructose could have a direct role in brain function or the metabolism of fructose systemically could lead to the release of factors affecting brain metabolism and function has not been explored.

In accordance to our findings that showed that a short-term (3 months) currant consumption reduces Aβ42 levels and neuroinflammation in 5xFAD mice, a previous study also showed that a short-term (2 months) administration of currants in a rat model of AD induced by intraperitoneal injection of aluminum chloride attenuates neuronal degeneration and abnormal histological architectures [14]. Phenolic (e.g., resveratrol, epigallocatechin gallate, quercetin, and kaempferol) and other compounds of Corinthian currants might participate in these beneficial effects. Such polyphenolic compounds have been shown to be able to cross the blood–brain barrier in in vitro studies or in in vivo studies by measurement of brain accumulation in rats following oral administration of polyphenols [68, 69] and therefore they have the potential to exert neuroprotective effects in the onset or during the progression of AD. Polyphenols, found in Corinthian currants [15, 20], have been shown to prevent cellular pathways related to inflammation, including the reduction of cytokine expression or secretion [22]. Resveratrol can elicit inhibitory effects on the release of inflammatory mediators and cytokines from several cells [70], while kaempferol can inhibit transcription factors responsible for the activation of inflammatory mediator genes [71]. Additionally, polyphenolic compounds of Corinthian currants have been shown to display anti-amyloidogenic effects, including reduction of Aβ and inhibition of Aβ oligomer formation [23–26]. Resveratrol can promote the intracellular degradation of Aβ and protect cells against Aβ-induced cytotoxicity [24, 72]. Furthermore, epigallocatechin gallate was found to decrease Aβ production [25], quercetin to inhibit Aβ oligomer formation and to decrease Aβ-stimulated apoptosis in neuronal cultures [27] and kaempferol to reduce the formation and aggregation of Aβ [26]. Moreover, anthocyanins that are found in Corinthian currants [21], were shown to prevent the oligomerization of Aβ and modulate the inflammatory responses in brain cells, while mice or rats receiving anthocyanins displayed a marked decrease in neuroinflammatory markers [73]. Therefore, the detailed evaluation of the beneficial effects of individual Corinthian currant components in AD animal models could be a further step towards the development of AD therapeutics.

Collectively, the studies in cell lines and animal models that display promising results for the effect of currants and their components in AD encourage the investigation of consuming currants in interventional studies with humans. Based on the current findings, any intervention with Corinthian currants or currant paste should be initiated in the early stages of disease pathogenesis and avoid the prolonged administration- a rough correlation between mouse and human life suggests that 6 mouse months are equivalent to 25 human years. Specifically, for targeting neuroinflammation in AD, animal and human studies indicate that anti-inflammatory treatments are more likely to exert beneficial effects at the early stages of disease. It has been proposed that at early stages a prevailing pro-inflammatory process can be treated and reversed, while at later clinical stages, the presence of sustained and excessive brain damage may reciprocally fuel a deregulated innate and adaptive immune response that renders anti-inflammatory treatments largely ineffective [74]. Furthermore, novel strategies that are being developed to reduce sugars in fruits, such as the use of probiotic bacteria (e.g., *Zymomonas mobilis*) that thrive in high-sugar environments and have been shown to reduce the glucose and fructose from fruit pastes [75], could be also utilized to reduce the sugar-concentration in currant paste as well.

Overall, our study shows that Corinthian currant consumption impacts neuroinflammation in a time-dependent manner in the 5xFAD mouse model of AD. Long-term intake of currants or glucose/fructose at low levels is shown to enhance neuroinflammatory responses in the brain of mice, despite the lack of effect in serum glucose or insulin levels. To the best of our knowledge, the impact of chronic intake of low levels of sugars on brain health and AD is largely unexplored and current findings create new questions on the effects and molecular mechanisms of chronic intake of natural sugar-containing products on brain function. However, the short-term intake of currants was found to exert beneficial effects by reducing the levels of AD-related pathogenic molecules in the 5xFAD mouse model of AD. Since synaptic and neuronal loss is largely irreversible in the late stages of AD pathogenesis, any disease-modifying dietary interventions should be considered early before the onset of severe neurodegeneration.

## Supplementary Information

Below is the link to the electronic supplementary material.

Supplementary file1 (DOCX 699 KB)

## Data Availability

The datasets supporting the findings of this study are available from the corresponding author on reasonable request.

## Abbreviations

Aβ: Amyloid‐β peptide
AD: Alzheimer’s disease
APP: Amyloid precursor protein
apoE: Apolipoprotein E
BDNF: Brain-derived neurotrophic factor
Con: Control
Cur: Currant
CSF: Cerebrospinal fluid
G/F: Glucose/fructose
GSK3β: Glycogen synthase kinase 3β
IL-1β: Interleukin 1β
PBS: Phosphate-buffered saline
NHS: Normal horse serum
TNFα: Tumor necrosis factor α
