# Supplementary material for: Temporal Pattern of Neuroinflammation Associated with a Low Glycemic Index Diet in the 5xFAD Mouse Model of Alzheimer’s Disease
Source: Mol Neurobiol. 2022 Sep 29;59(12):7303–22. doi: 10.1007/s12035-022-03047-3 (PMC9616770; doi:10.1007/s12035-022-03047-3)
Supplement: Supplementary file 1 — Supplementary file1 (DOCX 699 KB) [file 12035_2022_3047_MOESM1_ESM.docx]

**Supplemental Materials and Methods**

**Cell proliferation assay**

BV2 cells were plated on 96-well plates at a density of 5 x 10^3^ cells per well in RPMI complete medium. The next day, the cells were incubated in the absence or presence of raisin extract at concentrations 1 and 5 μg GAE/mL or glucose/fructose mixture at concentrations 2.6 and 12.6 mM, in serum-free medium for 24 and 48 h at 37^o^C. The final methanol concentration in each well was 0.68% (v/v). Cells before any incubation (0 h) and cells following incubations at 24 and 48 h were subjected to the 3-(4,5-dimethylthiazol-2-yl)-2,5-diphenyltetrazolium bromide assay (MTT) assay which measures only the *in vitro* living cells and indicates the incubation effects related to the number of viable cultured cells. Hence, cell media were aspirated and the cells were incubated in serum-free medium containing 0.65 mg/ml MTT (Sigma-Aldrich) for 3 h at 37 ◦C. Finally, the medium was removed, the dark blue formazan crystals formed by the cells were dissolved in DMSO, and absorbance was measured at 550 nm by an Infinite M200 plate reader (Tecan Group Ltd).

**Supplemental Figures**

**
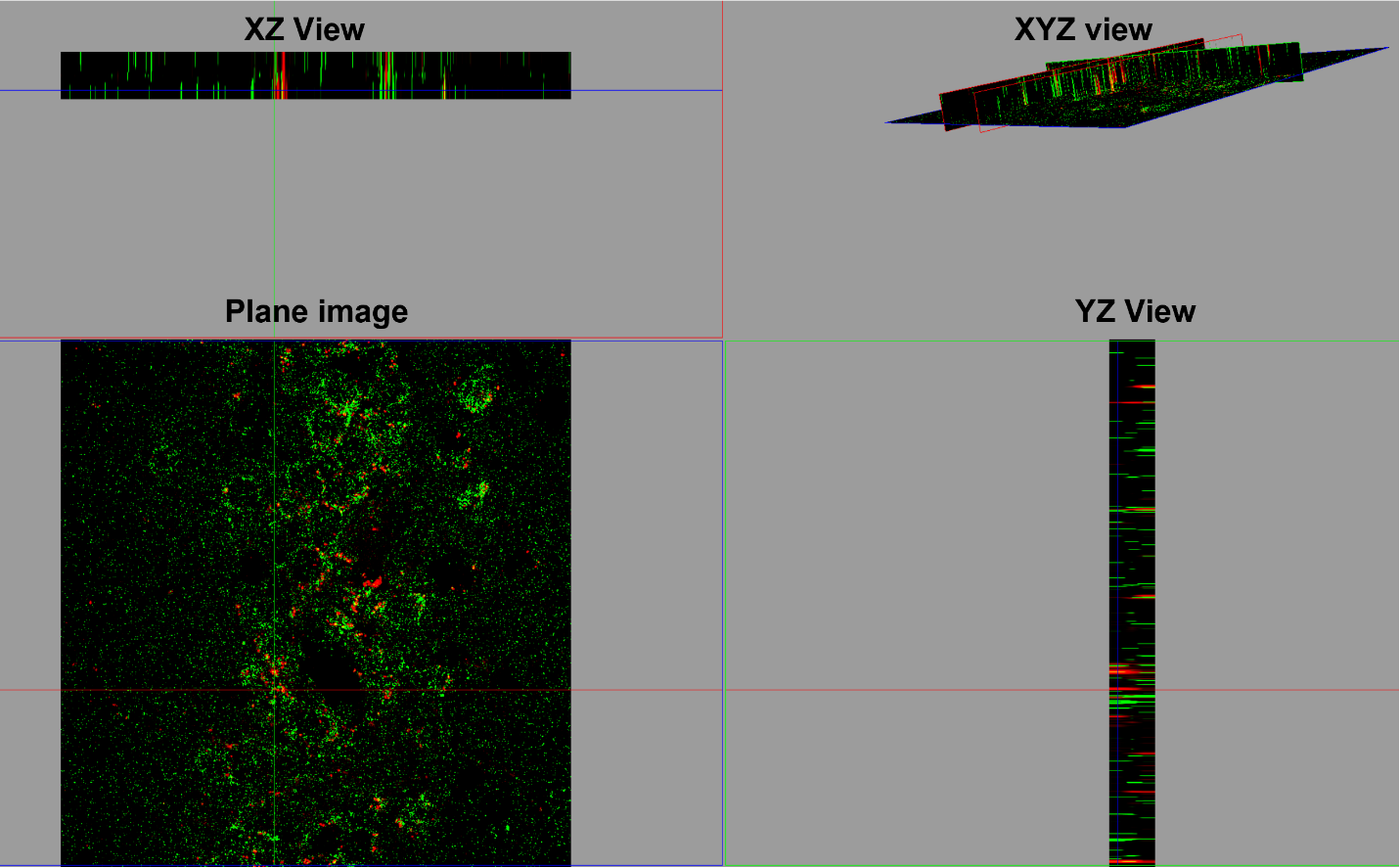
**

**Supplemental Figure 1:** Indicative confocal 3D-orthogonal views from CA3 layer of the G/F group of 5xFAD mice verifying the colocalization of TNFα^+^ (green) with CD11b^+^ cells (red). The green line indicates the X axis, the red line the Y axis and the blue one the z axis.

**
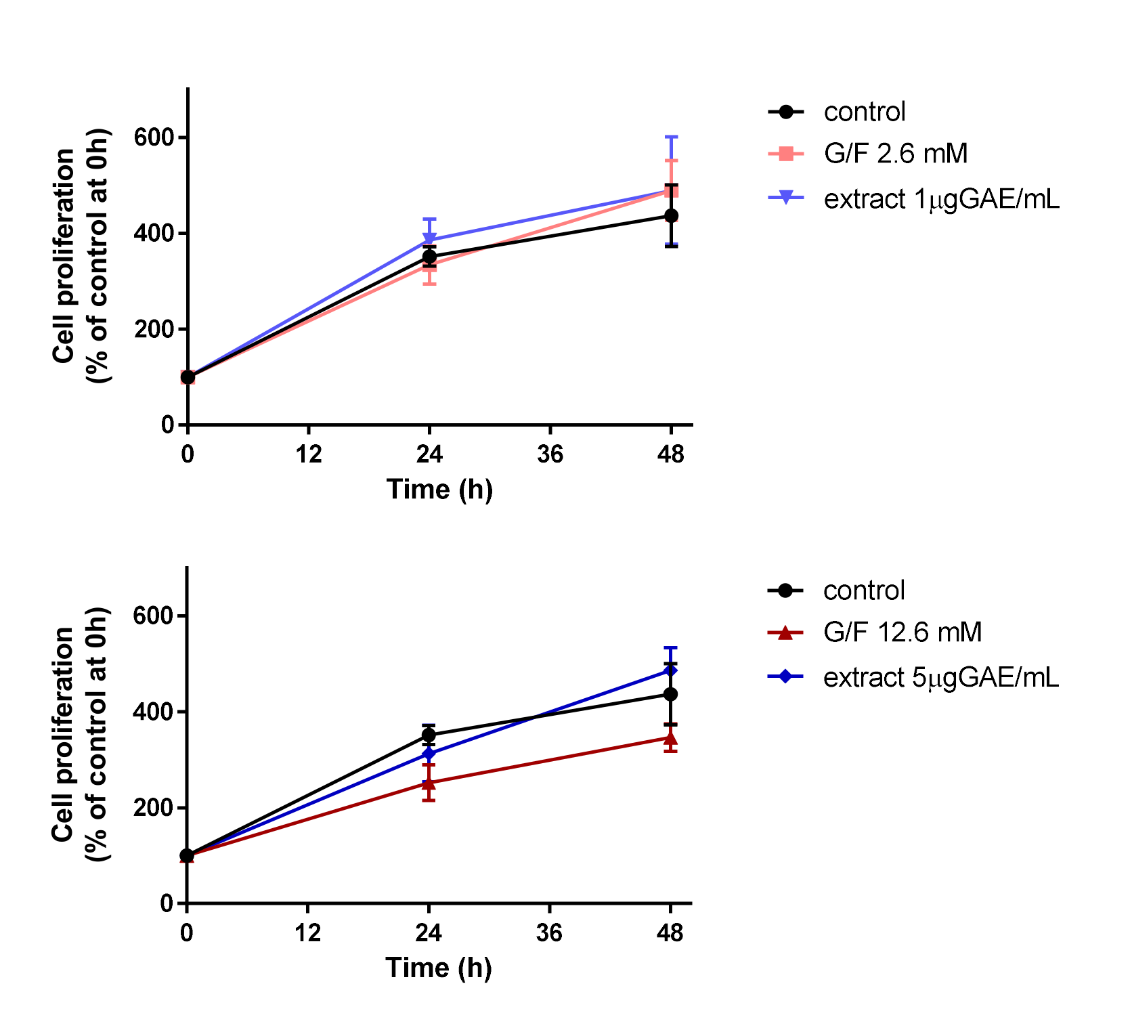
**

**Supplemental Figure 2:** Effect of raisin phenolic extract or glucose/fructose mixture in BV2 cell proliferation. BV2 cells were incubated in the absence or presence of raisin polar phenolic extract at concentrations of 1 μg GAE/mL (A) and 5 μg GAE/mL (B) or glucose/fructose (1:1 mol/mol) mixture at concentrations 2.6 mM (A) and 12.6 mM (B) that match the glucose/fructose concentration of raisin extract. At the incubation time points indicated, cells were harvested and subjected to the MTT assay. Cell proliferation was calculated as a percentage relative to values of control (untreated) cells at 0h (100%). Each point represents the mean ± SD from three independent experiments performed in duplicate.
